# Epidemiology of Pediatric Open Globe Injuries in a University Hospital in Thailand

**DOI:** 10.7759/cureus.19366

**Published:** 2021-11-08

**Authors:** Piangporn Saksiriwutto, Pariya Charuchinda, La-ongsri Atchaneeyasakul, Thammanoon Surachatkumtonekul, Pittaya Phamonvaechavan

**Affiliations:** 1 Ophthalmology, Siriraj Hospital, Mahidol University, Bangkok, THA

**Keywords:** retinal detachment, eye injury, open globe injury, visual impairment, epidemiology, pediatric

## Abstract

Objective: To describe the epidemiology, clinical characteristics, and clinical outcomes of pediatric traumatic open globe injuries and to determine the risk factors for poor visual outcome.

Methods: The medical records of patients aged younger than 15 years of age who were diagnosed with open globe injuries from January 2005 to December 2015 were retrospectively reviewed. The patients’ demographic data were collected, including age, sex, injury date, place of injury, mechanism of injury, cause of injury, and the activity related to the injury. Clinical data were recorded, including initial visual acuity (VA), wound size, wound location, associated ocular findings at presentation, and complications. The prognostic factors for a poor visual outcome were assessed.

Results: In total, 46 pediatric patients were included in this study. The mean age was 6.8 years old. Most patients were male (65.2%). The most common type of injury was penetrating injury (60.9%) and mostly occurred during playing (60.9%). Household appliances/furniture and scissors/knives were common causes of injuries (17.4%, 15.2%, respectively). Poor final VA worse than 6/60 was found in 17 patients (37%). Wound location and retinal detachment (RD) at the time of presentation were significant prognostic factors for a poor visual outcome according to the univariate analysis (p = 0.008, <0.001). Only wound location at zone II and III was found to be significantly correlated with poor final VA in the multivariate analysis (adjusted risk ratio (RR) = 2.87, 95% confidence interval (CI), 1.26-6.55, p = 0.012). Traumatic cataract was the most common associated injury (45.7%).

Conclusions: One-third of pediatric patients with open globe injuries had a poor visual outcome. Wound location at zone II and III significantly correlated with a poor visual outcome in pediatric open globe injuries. The parents and caregivers should be made aware of the seriousness of open globe injuries in order to prevent children from possible injuries.

## Introduction

In children, monocular visual impairment may disrupt binocular vision and affects visual development, which is very fragile, especially in infants and preschoolers. It can delay a child’s development, mental growth, and learning. In addition, amblyopia occurring after moderate to severe eye injuries may cause permanent visual loss in these children.

Ocular trauma is one of the most significant causes of monocular or even binocular visual loss in children [1], particularly in those who suffer from severe open globe injuries. The final visual outcome after complete treatment is different among the reported studies in each country. The average age varies from 7 to 11 years old, and the affected children are predominantly males [2-11]. The etiologies of the open globe injuries in children are different from those in adults. While adults usually suffer from work-related injuries [4], injuries in children mainly occur during playing and are commonly caused by sharp objects, e.g., a glass, a knife, or scissors [3,5]. Previous studies also found several prognostic factors that affected the final visual acuity (VA), including age, initial VA, wound location, wound size, and coinciding injuries, such as vitreous hemorrhage (VH), retinal detachment (RD), and endophthalmitis [3-6]. There are a few studies in the literature reporting the characteristics, visual outcome, and prognostic factors of open globe injuries in Thailand [8,9]; only the study by Choovuthayakorn et al. reported these in children from northern Thailand [9]. The epidemiology and characteristics of these patients could be different depending on geographical locations. Our study aims to investigate the epidemiology, visual outcomes, and prognostic factors of open globe injuries among pediatric patients who were treated at a tertiary medical center in central Thailand.

## Materials and methods

This retrospective descriptive study was conducted at Siriraj Hospital, Mahidol University. The protocol for this study was approved by the Siriraj Institutional Review Board (SIRB) (approval no. SI 503/2016). The medical records of patients under 15 years of age who were diagnosed with open globe injuries from January 2005 to December 2015 were retrospectively reviewed. Informed consent was waived by the IRB due to the retrospective nature of this study. We excluded patients who had a primary surgical procedure from another hospital and patients who had a follow-up period of less than three months. The included patients were divided into three subgroups based on age: 1-5, 6-10, and 11-15 years old. Open globe injuries were classified according to the Birmingham Eye Trauma Terminology (BETT) into four types: penetrating injury, perforating injury, intraocular foreign body (IOFB), and globe rupture [12].

The patients’ demographic data, including age, sex, injury date, place of injury, mechanism of injury, cause of injury, and activity related to the injury, were collected. Initial VA, wound size, wound location, and any associated ocular findings at presentation were recorded as clinical data. The primary surgical procedure, subsequent surgical procedures, and complications were also recorded. Wound location was defined into three zones, according to a previous study [13]; zone I (within the cornea), zone II (limbus and sclera 5 mm posteriorly), and zone III (posterior to zone II). If the injury involved multiple zones, it was defined by the most posterior extension. The presenting parameters were analyzed for their effect on the final visual outcome.

Final VA was assessed from the last visit, at least three months after the injury. The fixation and following behavior in preverbal children was considered as hand motion [12]. A poor visual outcome was defined as a final VA of less than 6/60.

Descriptive data were reported using mean, median, and percentage as appropriate. The association between each factor and a poor visual outcome was analyzed using the Pearson chi-square test and crude relative risk. A p-value of less than 0.05 was considered statistically significant. Factors with a p-value less than 0.1 from the univariate analysis were entered into a multivariate analysis using a log-link binomial regression model, and the results were reported with the adjusted relative risk, 95% confidence interval (CI), and p-value. The statistical analysis was performed using STATA version 16.0 (STATA Corp., College Station, TX, USA) and Statistical Package for the Social Sciences (SPSS) version 18.0 (SPSS Inc, Chicago, IL, USA).

## Results

A total of 46 pediatric patients (46 eyes) diagnosed with open globe injuries between January 2005 and December 2015 were included in the study analysis. Bilateral eye injury was not found in this study. The patients’ demographic data are shown in Table 1. The mean age of the patients was 6.8 ± 3.5 years (range 1-13 years), and around 80% (n=27) were under 10 years old. The majority of the patients were male (n=30, 65.2%) and the male to female ratio was approximately 1.9: 1. Almost 70% of the patients (n=32) had an initial VA worse than 6/19. The initial VA could not be evaluated in eight patients due to poor cooperation. Over half of the injuries occurred at home (n=31, 67.4%), followed by at school (n=8, 17.4%), while the place of injury could not be identified in seven patients. Penetrating injury was the most common type of injury (n=28, 60.9%), followed by globe rupture (n=15, 32.6%) and IOFB (n=3, 6.5%). No perforating injury was found in this study. The injuries mostly occurred during playing (n=28, 60.9%) followed by during a daily activity at home (n=7, 15.2%). Most injury’s location occurred in zone I (n=34, 74.%). Twenty-six patients (56.5%) had a wound size larger than 5 mm.

**Table 1:** Clinical data of the pediatric open globe injury patients VA = visual acuity, IOFB = intraocular foreign body

Parameters	Total pediatric open globe injury	Good visual outcome group	Poor visual outcome group	p value
	(n=46)	(n=29)	(n=17)	
Age (years)				0.422
0-5	19 (41.3%)	11 (37.9%)	8 (47.1%)	
6-10	19 (41.3%)	14 (48.3%)	5 (29.4%)	
11-15	8 (17.4%)	4 (13.8%)	4 (23.5%)	
Male gender	30 (65.2%)	20 (69.0%)	10 (58.8%)	0.486
Place				0.288
Home	31 (67.4%)	18 (62.1%)	13 (76.5%)	
School	8 (17.4%)	7 (24.1%)	1 (5.9%)	
Unknown	7 (15.2%)	4 (13.8%)	3 (17.6%)	
Mechanism of injury				0.274
Penetrating	28 (60.9%)	20 (69.0%)	8 (47.1%)	
Rupture	15 (32.6%)	7 (24.1%)	8 (47.1%)	
IOFB	3 (6.5%)	2 (6.9%)	1 (5.9%)	
Initial VA				0.088
VA < 6/19	32 (69.6%)	21 (72.4%)	11 (64.7%)	
VA ≥ 6/19	6 (13.0%)	6 (20.7%)	0	
Wound location				0.018
Zone I	34 (74.0%)	25 (86.2%)	9 (53.0%)	
Zone II or III	12 (26.0%)	4 (13.8%)	8 (47.0%)	
Wound size				0.016
0-5 mm	20 (43.5%)	16 (55.2%)	4 (23.5%)	
>5 mm	26 (56.5%)	13 (44.8%)	13 (76.5%)	

The causes of injuries varied regarding the activities and places of the injuries. Household appliance/furniture and sharp objects (scissors/knife) were the two most common causes of injuries (17.4%,15.2% respectively). Causes of injuries are shown in Table 2. Two of the 46 patients had been attacked by animals, namely a bird, and a chicken while playing with these animals. As for surgical repair, most of the patients underwent surgeries within 24 hours after the injuries and the median time was 10 hours (range 3.5-264 hours). All the patients received topical antibiotics, and 43 out of the 46 patients also received systemic antibiotics. One outlier patient, a 5-year-old girl, presented to the hospital 11 days after the injury with an unknown cause of injury, and unnoticed by the family members. The diagnosis in this girl was a penetrating injury in zone I with RD and endophthalmitis. Her initial VA was hand motion, which was not improved despite having corneal wound repaired and vitrectomy. The follow-up period for the 46 patients ranged from three months to 10 years, and the median time was 32 months.

**Table 2:** Causative objects of pediatric open globe injuries

Objects	Total pediatric open globe injury
	(n=46)
Scissors/knife	7 (15.2%)
Wooden object	4 (8.7%)
Household appliance/furniture	8 (17.4%)
Glass/bottle	3 (6.5%)
Darts/spinning top	4 (8.7%)
Stationary	4 (8.7%)
Animal	2 (4.3%)
Firework	2 (4.3%)
Stone/clod	3 (6.5%)
Wire/Steel	2 (4.3%)
Others	4 (8.7%)
Unknown	3 (6.5%)

Table 3 demonstrates the univariate and multivariate analysis of the factors that affected a poor final visual outcome. At a three-month follow-up, 29 patients (63%) had a good final VA, while 17 patients (37%) had a final VA worse than 6/60. The present study showed a significant association between wound location and RD with the final visual outcome in univariate analysis (risk ratio (RR) 2.52, 95% CI 1.27 - 5.01, p = 0.008 and RR 3.07, 95% CI 2.00 - 4.27, p < 0.001, respectively). Most of the patients with wound location in zone I (25/34, 73.5%) had a good final VA, conversely, around 67% of the patients (8/12) whose wound was located in zone II or III had a poor final VA. Unfortunately, there were only three patients with RD found in this study, and all of them resulted in a poor visual outcome, thus we could not include RD in our regression model for multivariate analysis because it might make the model unstable due to the scarcity of this event in our cohort. Wound size, which was one of our interesting factors, was included in the multivariate analysis. The results showed no correlation between wound size and visual prognosis. Only wound location at zone II and III was found to be the factor that statistically correlated with the poor final VA in the multivariate analysis (RRadj = 2.87, 95% CI, 1.26 - 6.55; p = 0.012).

**Table 3:** Factors affecting the visual outcome (univariate and multivariate analysis) VA = visual acuity, IOFB = intraocular foreign body

Parameters	Univariate analysis			Multivariate analysis		
	RR	95% CI	p value	Adjusted RR	95% CI	p value
Age (years)						
0–5	1	-	-	-	-	-
6–10	0.63	(0.25 -1.57)	0.316			
11–15	1.19	(0.05 - 2.84)	0.699			
Initial VA < 6/19	-	-	0.150	-	-	-
Mechanism						
Penetrating	1	-	-			
IOFB	1.17	(0.21 - 6.41)	0.859	-	-	-
Rupture	1.87	(0.88 - 3.96)	0.104			
Wound location at zone II or III	2.52	(1.27 - 5.01)	0.008	2.87	(1.26 - 6.55)	0.012
Wound size > 5 mm	2.50	(0.96 - 6.51)	0.060	2.23	(0.74 - 6.74)	0.156
Associated findings						
Hyphema	1.25	(0.58 - 2.70)	0.575	-	-	-
Traumatic cataract	0.50	(0.21 - 1.18)	0.113	-	-	-
Vitreous hemorrhage	0.89	(0.27 - 2.95)	0.847	-	-	-
Retinal detachment	3.07	(2.00 - 4.27)	<0.001	-	-	-
Endophthalmitis	1.40	(0.48 - 4.04)	0.534	-	-	-

Traumatic cataract was the most common finding associated with open globe injuries (n=21, 45.7%), in which 17 out of 21 patients (81%) underwent cataract surgery during the primary globe repaired or as a subsequent operation. The other associated findings were hyphema (n=14, 30%), VH (n=6, 13%), RD (n=3, 7%), and endophthalmitis (n=4, 9%). The univariate analysis, presented in Table 3, showed no significant correlation between the associated injuries (hyphema, traumatic cataract, VH, and endophthalmitis) and the final visual outcome. RD was found in three patients, only one patient underwent retinal surgery as a second operation, the other two patients had very severe open globe injuries where the retina could not be repaired. Two of them ended up with VA of no light perception and another one had VA at hand motion.

Endophthalmitis was found in four patients and all the patients received both systemic and intravitreal antibiotics. Two patients had a good final VA (6/6 and 6/15) whose wound locations were at the corneoscleral area in zone I with no associated injury. Two patients had a poor final VA (no light perception and hand motion) and their lacerated wounds were located at the center of the cornea. Vitreous cultures were negative in all four cases. Polymerase chain reaction for bacteria was positive for Streptococcus spp. in one patient, who had a delayed diagnosis (11 days after the injury) with an unknown cause of injury. This patient had not only endophthalmitis but also RD at presentation, resulting in phthisis bulbi and a final VA of no light perception. Approximately, 50% of the patients underwent more than one operation. No patients in this study required enucleation.

In this study, 10 patients (21.7%) developed strabismus in the traumatized eye that had VA worse than the fellow eye. Seven patients (15.2%) with poor final VA developed phthisis bulbi.

## Discussion

In this study, open globe injury was found more commonly in males, and mostly in children under 10 years old. Approximately 60% of our patients had penetrating injuries, and none suffered from a perforating injury. Seventeen patients (37%) had a final VA worse than 6/60, and according to multivariate analysis, the posterior wound location was the only factor that had a significant correlation with a poor final visual outcome. 

Similar to other studies worldwide, our study showed that boys were more susceptible to suffer from open globe injuries than girls [2-11]. The male predisposition could be explained by the boy’s tendency to involve in more dangerous activities that make them prone to the risk of experiencing a traumatic eye injury. Most of our patients were relatively young (under 10 years old), consistent with the results from previous reports [5,10]. In contrast, the study from northern Thailand by Choovuthayakorn et al. [9] revealed most of their patients were older at ages between 11-16 years old. The variability among age groups may be the result of the differences in nurturing, lifestyle, and caregiver’s education. Our study also demonstrated that injuries in children majorly arose at home (67.4%), as opposed to adults whose injuries commonly occur at a workplace [4,8]. Interestingly, only 17.4% of the children’s eye injuries in our study happened at school despite children spending more time at school than at home, similar to the result of Sul et al. [6] and Tok et al. [10] that showed most of the injuries occurred at home and there were a little number of the injuries at school (4.4% and 8.5%, respectively). This lower rate of injury at school may be due to the presence of caregivers to monitor the children’s activities and relatively more difficult access to hazardous objects at school.

In our study, the injuries mainly occurred while the patients were playing (60.9%), which corresponds to the result of Choovuthayakorn et al. [9]. However, they found that wooden object was the most common object that caused the injuries, contrasting to our study in which household appliance/furniture and scissors/knife were the major culprits. Other objects such as glasses, darts, stationary, firework, stone, wire, and even animals have been reported to cause open globe injuries [5-7,11]. This study found two cases (4.3%) where the injuries had been caused by an animal attack, which was also previously reported by Li et al. (1.4%) [11]. The differences in the cause of injury may be related to the patients’ age, activities, and place of the injuries, which vary by region and country. Due to children’s active and curious nature, parents, teachers, and caregivers should be cautious and pay close attention to their children, especially those under 10 years old. All household appliances and hazardous objects should be kept out of the reach of young children, and pets should also be closely monitored, as they can potentially cause a very serious injury.

Visual assessment in young children is very challenging, especially in children suffering from eye injuries. Almost 70% of the children in our study had an initial VA worse than 6/19. This assessment may not be reliable given varying children’s cooperation during the examination. We were unable to assess initial VA in almost one-fifth of our patients (17%). Although the initial VA was reported to be one of the predictive factors of a poor visual outcome in many studies [4,6,10,11], our study did not show an association between the initial VA and the final VA. It may be a result of unreliable initial VA of the children who suffered from the injuries, which tended to be underestimated. Another possible explanation is we have a small number of patients with good initial VA in our study. Similar to other studies [3,4,6,9-11], the penetrating injury was the most common type of open globe injury followed by rupture and IOFB. In our study, 70% of the patients with penetrating injury had a final VA better than 6/60, and approximately half of the rupture injuries had a final VA worse than 6/60; however, there was no statistically significant correlation between the final visual outcome and the type of injuries. It may possibly be from the small number of patients in each type of injury resulting in the insignificant correlation between these two factors. Lesniak et al. [3] and Tok et al. [10] reported that zone I was the most common location of the injuries, similar to this study. The more posterior the wound extension, the more severe the injuries are due to vital organs being damaged. Bunting et al. [5] reported that a retrolimbal wound location (zone II, III) was one of the independent predictive factors for the final visual outcome along with an age under 5 years old, blunt injury, a wound length larger than 6 mm, VH, and RD. In this study, the multivariate analysis showed only wound location at zone II, III to be a significant prognostic factor for a poor visual outcome.

The associated finding in children who had open globe injuries that was mostly observed in the present study was traumatic cataract (46%). Approximately 81% of patients had cataract surgery among these 76% of patients with traumatic cataracts had a final VA better than 6/60. However, traumatic cataracts had no significant correlation with the final VA.

RD was found in three patients and all of them had poor final VA. RD was found to be a significant prognostic factor for poor visual outcome in pediatric open globe injuries in several previous studies [5,6,9,10]. Unfortunately, we excluded RD from the multivariable analysis because of a small number of patients with RD. However, we believed that RD is a potential risk factor for poor visual outcome since the attachment of the neurosensory retina and retinal pigment epithelium was directly correlated with the visual function and it was previously reported by many studies mentioned above as one of the predictors of poor visual outcome.

Endophthalmitis is one of the most serious complications of open globe injury. Sul et al. [6] reported a detection rate of endophthalmitis of 12.1% and found it was statistically significantly related to a poor visual outcome. Endophthalmitis was found in four patients in the present study of whom, two had a good final VA, and the other two had a poor final VA; therefore there was no significant difference found in the statistical analysis between endophthalmitis and a poor visual outcome, consistent with a previous report [9]. This result may be due to our small sample size. Additionally, several factors may result in ocular infection after open globe injuries, such as contaminated wound, time to the first operation, and time to the first antibiotic administration. The differences in these factors may affect the rate of endophthalmitis in each study.

The severe open globe injuries can lead to profound visual loss, which is difficult to treat. In severe injury cases, phthisis bulbi can develop and affect patients and families both physically and psychologically. Our study showed that about 15% had phthisis bulbi as a final complication from open globe injuries.

The limitations of our study are the small sample size, the retrospective nature, and the relatively short follow-up period. This study had a short follow-up period as most patients were referred cases, so once they had received complete treatment, they were referred back to ophthalmologists at their primary care hospitals, and some patients were lost to follow-up. The true long-term final VA might differ from the documents we had in some patients. A larger study recruiting patients from multi-centers in varying regions would help increase the study population, which might increase the reliability of the study results.

## Conclusions

Open globe injury occurs mostly in male children under 10 years of age during playing at home. The final visual outcome can be unsatisfactory despite appropriate treatments. The posterior wound location was the only predictive factor that correlated with the poor final VA. Poor final visual outcomes in severe cases not only affect the children’s well-being but also their families as these children will require extensive care from their parents or caregivers. Therefore, good and responsible caring by parents and related caregivers is crucial. Public awareness about eye injury, its mechanism, and preventive measures should be raised in order to decrease the incidence of traumatic eye injuries among children. 
